# EXCLUSION FROM CIVIC ENGAGEMENT IN TWO DISADVANTAGED NEIGHBORHOODS IN BRUSSELS, BELGIUM: A LIFE COURSE PERSPECTIVE

**DOI:** 10.1093/geroni/igad104.1674

**Published:** 2023-12-21

**Authors:** Bas Dikmans, Liesbeth De Donder, Dury Sarah, Rodrigo Serrat

**Affiliations:** Vrije Universiteit Brussels, Brussels, Brussels Hoofdstedelijk Gewest, Belgium; Vrije Universiteit Brussel (VUB), Brussels, Brussels Hoofdstedelijk Gewest, Belgium; Vrije Universiteit Brussel, Brussels, Brussels Hoofdstedelijk Gewest, Belgium; University of Barcelona, Barcelona, Catalonia, Spain

## Abstract

Although research into older people’s civic engagement has come a long way in the last decades, there are still some blind spots on this scholarship. Notably, the influence of neighbourhood features on in- or exclusion from civic activities, as well as the multifaceted nature of civic engagement and the dynamics of such processes across the life course have been scarcely addressed. Therefore, this research focuses on the life course trajectories of older people in two socioeconomically disadvantaged neighborhoods in Brussels, Belgium, in order to understand their inclusion and exclusion from multidimensional forms of civic engagement, including formal volunteering, informal helping behaviours, memberships in associations, political participation and digital civic engagement. We conducted forty semi-structured interviews supported by life diagrams that focused on the changes of in- and exclusion from multidimensional civic engagement throughout the life course. The life stories of these older residents are thematically and narratively analyzed. The outcome of this study underlines the importance of a life course perspective in understanding older people’s experiences of inclusion and exclusion from multidimensional civic engagement. The findings will be used for informing policy recommendations to foster multidimensional civic engagement for older people living in disadvantaged communities.

